# Management Strategies for Refractory Esophageal Varices

**DOI:** 10.1002/deo2.70155

**Published:** 2025-06-19

**Authors:** Keita Maki, Hiroaki Haga, Yoshiyuki Ueno

**Affiliations:** ^1^ Department of Gastroenterology Yamagata University Faculty of Medicine Yamagata Japan

**Keywords:** esophageal varices bleeding, internal medicine treatment, interventional radiology, refractory bleeding, surgical therapy

## Abstract

Refractory esophageal varices that are difficult to control or unresponsive to endoscopic treatment remain a major clinical challenge in the management of portal hypertension. This review provides a comprehensive overview of treatment strategies for these cases, along with a comparative analysis of the American Association for the Study of Liver Diseases, Baveno VII, and Japanese clinical practice guidelines. Treatment approaches are classified into four domains: endoscopic therapy, interventional radiology (IVR), surgical procedures, and internal medicine‐based strategies. In recent years, clinical practice has shifted from traditional surgical interventions and transjugular intrahepatic portosystemic shunt (TIPS) to minimally invasive IVR techniques, such as partial splenic embolization, percutaneous transhepatic obliteration, and transileocolic vein obliteration, often combined with endoscopic methods. In Japan, where TIPS is not routinely performed due to limited availability and lack of insurance coverage, these alternative IVR procedures are more commonly utilized. Differences among regional guidelines highlight the need for adaptable treatment strategies based on local resources and institutional expertise. Effective management of refractory cases requires multidisciplinary collaboration among gastroenterologists, interventional radiologists, and surgeons. This review emphasizes the importance of integrating international evidence with local clinical practice to develop a tailored, team‐based approach that improves outcomes in patients with complex variceal disease.

## 1 Introduction

The rupture of esophageal varices is one of the most fatal complications of liver cirrhosis [1]. Esophageal varices are present in approximately 30% of patients at the time of liver cirrhosis diagnosis [2]. The lifetime incidence of esophageal varices in cirrhosis patients is high, ranging from 80% to 90% [3]. Additionally, approximately 42% of patients with Child‐Pugh grade A cirrhosis and 72% of those with Child‐Pugh grade B/C cirrhosis have gastroesophageal varices [2]. In patients without preexisting varices, the incidence of new varices is expected to exceed 5% per year [4, 5, 6, 7].

Endoscopic treatment is the primary approach for managing esophageal varices, whether for cases of rupture or elective treatment [1, 8–11]. However, endoscopic treatment can be challenging in certain situations, such as when varices are large and endoscopic variceal ligation (EVL) alone is insufficient, when suction during EVL is difficult, or when sclerotherapy fails due to high internal pressure within the varices. We previously reported that endoscopic treatment becomes particularly difficult when esophageal varices exceed 15 mm in diameter, as EVL may not effectively occlude blood flow [12].

Refractory esophageal varices are defined as: 1) varices that are difficult to control endoscopically for hemostasis, and 2) varices that do not show regression after endoscopic treatment. In this study, we review treatment approaches for esophageal varices that are difficult to manage endoscopically, categorizing them into interventional radiology (IVR), surgical treatment, and medical treatment, based on the American Association for the Study of Liver Diseases (AASLD) and Baveno VII guidelines, as well as previous reports on refractory esophageal varices (Table 1). Additionally, we summarize cases of esophageal varices that are challenging to treat endoscopically and discuss strategies for managing refractory esophageal varices.

**TABLE 1:** Treatment approaches for esophageal varices include endoscopic treatment, interventional radiology, surgical therapy, and internal medical treatment.

*Endoscopic treatment* Endoscopic variceal ligation (EVL) Endoscopic injection sclerotherapy (EIS) Endoscopic therapy with tissue adhesives (ETA)
*Interventional radiology* ✓ Portal decompression Transjugular intrahepatic portosystemic shunt (TIPS) Partial splenic embolization (PSE) ✓ Collateral embolization Percutaneous transhepatic obliteration (PTO) Trans‐ileocolic vein obliteration (TIO)
*Surgical therapy* ✓ Direct surgery Hassab operation, Esophageal transection, etc. ✓ Shunt surgery Selective shunt (e.g., Distal splenorenal shunt [DSRS]) Systemic shunt (e.g., Portacaval shunt [PCS])
*Internal medicine treatment* Self‐expandable metal stents Balloon tamponade Non‐selective β‐blockers (NSBBs) (e.g., carvedilol, propranolol, nadolol) Vasoactive agents (e.g., octreotide, somatostatin, terlipressin)

### 1.1 Endoscopic Treatment

Johnston first reported endoscopic injection sclerotherapy (EIS) in 1973 [13], followed by Takase's report of EIS in Japan in 1978 [14]. Stiegmann developed EVL in 1988, and Yamamoto et al. introduced it to Japan [14, 15]. The characteristics of endoscopic treatments for esophageal varices, including EVL (the main endoscopic treatment worldwide), EIS, and endoscopic therapy with tissue adhesives (ETA), are described below (Table 2).

**TABLE 2:** Characteristics of main endoscopic treatment for esophageal varices.

	EVL	EIS	ETA
Advantages	✓ First‐line treatment for esophageal varices rupture worldwide. ✓ No bleeding from puncture or side effects from sclerosing agents. ✓ Can be safely performed even in patients with severe liver dysfunction or renal impairments.	✓ Lower recurrence rate of esophageal varices than EVL due to its effect on blocking blood supply routes. ✓ Widely used as an elective treatment, especially in Japan.	✓ Considered when hemostasis is difficult to achieve with EVL. ✓ Demonstrates excellent hemostatic effects.
Disadvantages	✓ Aspiration can be difficult or unsuccessful in patients with prior variceal treatment. ✓ Aspiration can also be challenging in cases of large varices, such as pipeline esophageal varices. ✓ EVL alone results in a higher recurrence rate than EIS due to limited obstruction of blood supply.	✓ Emergency hemostasis has a higher rebleeding rate and complications than EVL. ✓ May cause liver dysfunction or renal damage.	✓ More technically demanding than EVL or EIS due to the rapid hardening of the adhesive, requiring significant operator experience. ✓ Not approved for use in Japan for the treatment of esophageal varices.

Abbreviations: EIS, endoscopic injection sclerotherapy; ETA, endoscopic therapy with tissue adhesives; EVL, endoscopic variceal ligation.

**FIGURE 1:**
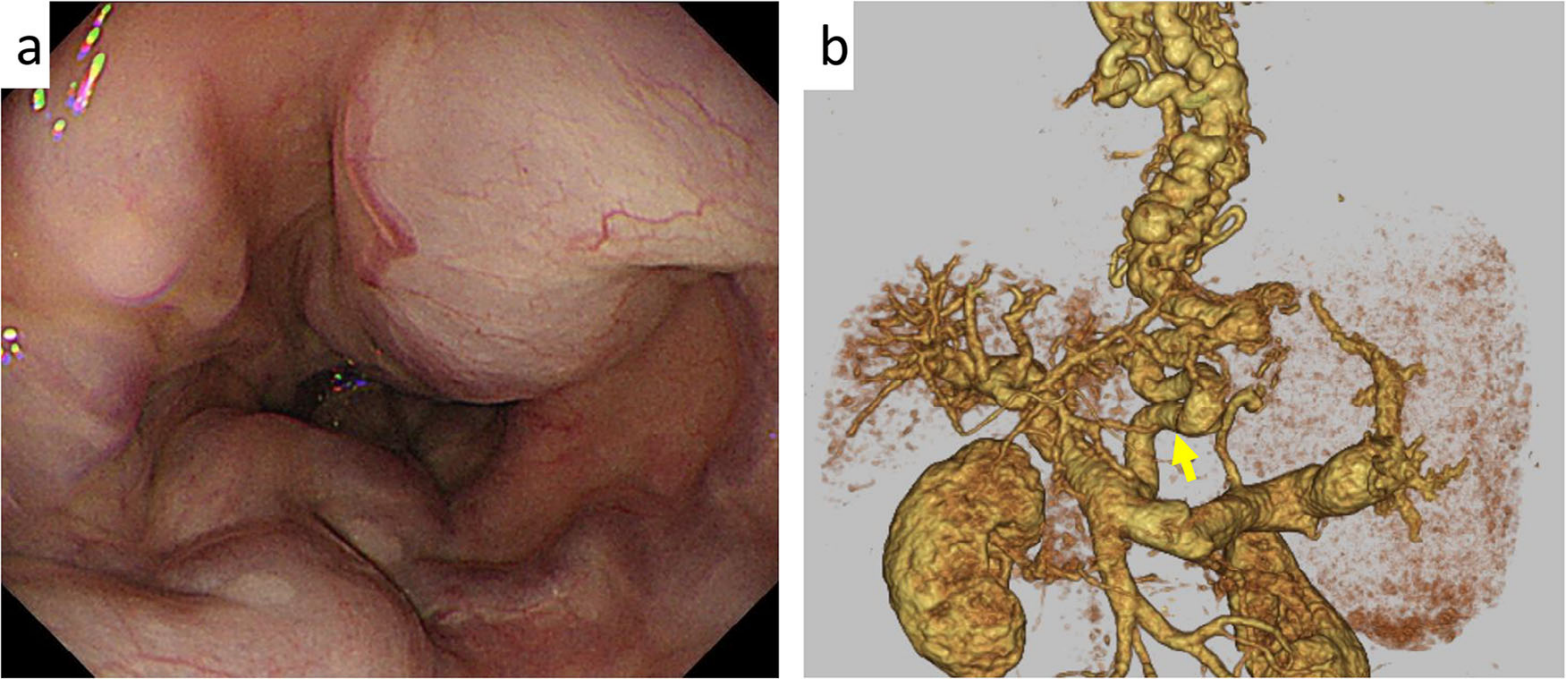
Images of pipeline esophageal varices: (a) Endoscopic images of pipeline esophageal varices. (b) 3D‐CT images of pipeline esophageal varices. Pipeline esophageal varices run from the left gastric vein through the gastric cardia and gastroesophageal junction to the middle and upper esophagus (arrow).

EVL is the first‐line treatment for ruptured esophageal varices [8, 16]. It achieves hemostasis in approximately 90% of active variceal bleeding cases [17]. However, in some patients, endoscopic control is difficult, particularly when EVL is insufficient due to large varices, such as pipeline esophageal varices (Figure 1a), or when esophageal mucosal fibrosis from previous endoscopic treatment prevents variceal suction into the O‐ring [12]. Pipeline esophageal varices are a distinct type of varices, characterized by a “pipeline‐like” appearance in portal vein imaging, which shows the varices running from the left gastric vein through the gastric cardia and esophagogastric junction to the middle and upper esophagus, without passing through the bamboo blind vessels of the lower esophagus (Figure 1b).

EIS occludes the blood flow that supplies the esophageal varices with a sclerosing agent. This is the reason why EIS shows a lower recurrence rate of gastroesophageal varices than EVL [18, 19]. Therefore, EIS is selected as the first choice of prophylactic variceal treatment in Japan.

Alternatively, EIS is said to have a higher rebleeding rate and complications than EVL in emergency hemostasis.^1^ Additionally, when compared with EVL, EIS has been linked to a higher risk of complications, including pleuropulmonary complications, bleeding, and infection [20].

EIS has been used to treat esophageal varices through two approaches: extravariceal and intravariceal sclerotherapy. Additionally, two types of sclerosants are commonly used in EIS—ethanolamine oleate (EO) and polidocanol. In Japan, EO is widely used for intravariceal sclerotherapy due to its greater ability to promote thrombus formation compared to polidocanol [21]. However, intravariceal sclerotherapy is technically demanding and requires considerable time and experience [22].

ETA offers a stronger hemostatic effect and is considered when hemostasis cannot be achieved with EVL. However, due to the rapid hardening of tissue adhesives, ETA is technically more challenging than EVL or EIS and requires procedural experience (Table 2). Although EVL and EIS are the primary endoscopic treatments for esophageal varices, the Baveno VII guidelines recommend ETA as a secondary option for ruptured varices.

In many countries, *N*‐butyl‐2‐cyanoacrylate (NBCA) is approved for ETA of esophageal and gastric (fundal) varices. However, in Japan, ETA is not yet approved for use in esophageal varices. NBCA polymerizes and hardens immediately upon contact with blood, enabling rapid control of active bleeding [23]. High rates of active bleeding control have been reported by Ljubičić et al. [24], Maluf‐Filho et al. [25], and Elsebaey et al. [26]. A retrospective study by Liu et al. showed no significant difference in hemostasis rates between EVL and ETA [27]. Therefore, ETA is a safe and effective option for controlling bleeding without interfering with subsequent variceal ligation [28]. Although ETA has demonstrated effectiveness in the treatment of esophageal varices in Japan [29], its clinical use remains limited. ETA is mainly considered a rescue therapy for immediate bleeding control or for cases that are difficult to manage with standard endoscopic procedures [30].

For emergency treatment, EVL is the first‐line option in both Japan and the West. However, for elective treatment, Japan primarily uses both EIS and EVL, whereas Western guidelines (AASLD, Baveno VII) recommend EVL in combination with non‐selective β‐blockers (NSBB). Although the AASLD and Baveno VII guidelines define indications for endoscopic examination and follow‐up strategies, the Japan guidelines lack such specific recommendations (Table 3).

**TABLE 3:** Emergency and elective endoscopic treatments for esophageal varices in Japanese, the American Association for the Study of Liver Diseases (AASLD), Baveno VII guidelines.

	Japan	AASLD	Baveno VII
Main emergency treatments	EVL	EVL	1st EVL, 2nd EIS, ETA, HP
Elective treatment indication (High‐Risk EV)	Medium or large EV, or EV with red color signs	Moderate or large varices, or any size varices with red wale marks, or patients with CTP‐C	Medium or large EV, or small EV with red wale marks
Main elective treatments	EIS, EVL ✓ If none of the following criteria are met, EIS is performed. ✓ If any of the following criteria are met, EVL is performed. CTP‐C T‐Bil ≥ 4 mg/dL Complication of advanced liver cancer (portal vein tumor thrombosis, Vp3/Vp4). ✓ Consolidation therapy (AS/APC) may be added after EIS/EVL.	EVL, NSBB If high‐risk varices are small, NSBB is selected. If high‐risk varices are large, both NSBB and EVL are possible. NSBBs (including carvedilol) are preferred due to their benefits beyond variceal hemorrhage prevention. Carvedilol is recommended as the preferred NSBB for the treatment of PH in patients with cirrhosis.	EVL, NSBB ✓ Carvedilol is recommended as the first‐choice NSBB.
Indications for endoscopic examination and guidelines for follow‐up	✓ No clear statement is provided in Japanese guidelines.	✓ Endoscopy is mainly for patients contraindicated for, intolerance to, or not taking NSBB. ✓ Follow‐up intervals vary based on: Compensated cirrhosis and CSPH or decompensated cirrhosis, Presence or absence of varices, Status of underlying disease (controlled or uncontrolled).	✓ Endoscopy is mainly for patients with decompensated ACLD and and LSM ≥ 20 KPa or platelet count ≤ 150 × 10^9^/L, or for compensated cirrhosis patients not candidates for NSBBs. ✓ The follow‐up intervals vary based on: 1. Presence or absence of esophageal varices, 2. Liver disease activity status (quiescent or ongoing risk factors).

Abbreviations: ACLD, advanced chronic liver disease; APC, argon plasma coagulation; AS, aethoxysklerol; CSPH, clinically significant portal hypertension; CTP‐C, Child‐Turcotte‐Pugh grade C; EIS, endoscopic injection sclerotherapy; ETA, endoscopic therapy with tissue adhesives; EV, esophageal varices; EVL, endoscopic variceal ligation; HP, hemostatic powders; LSM, liver stiffness measurement; NSBB, non‐selective β‐blockers; PH, portal hypertension; T‐Bil, total bilirubin; Vp, vascular permeation.

In the AASLD and Baveno VII guidelines, the hepatic venous pressure gradient (HVPG) is routinely used as the gold standard for diagnosing clinically significant portal hypertension [1, 17]. Controlling portal venous pressure is the central focus of Western treatment strategies for esophageal varices. In contrast, this concept is not thoroughly emphasized in the Japanese guidelines. This difference explains why NSBB is the main treatment in Europe and the United States. It may also reflect the higher cost of endoscopic examinations in the West compared to Japan, due to differences in insurance systems and healthcare access.

Detailed emergency hemostasis and secondary prevention procedures (Figure S1), as well as primary prevention strategies (Figure S2), according to the Japanese, AASLD, and Baveno VII guidelines, are provided below.

In Japanese guidelines, EVL or a Sengstaken–Blakemore (SB) tube is used for acute esophageal variceal rupture, while EIS or EVL is performed for secondary prevention, followed by consolidation therapy with aethoxysklerol or argon plasma coagulation. Endoscopic procedures account for the majority of variceal treatments (Figure S1) [9, 10].

In contrast, the AASLD and Baveno VII guidelines recommend EVL as the first‐line treatment for esophageal variceal rupture (referred to as endoscopic band ligation in the guidelines but hereafter referred to as EVL). For secondary prevention, NSBB and EVL are recommended in the AASLD and Baveno VII guidelines. Additional procedures, such as intrahepatic portosystemic shunts (pre‐emptive intrahepatic portosystemic shunt (transjugular intrahepatic portosystemic shunt [TIPS]) and transplants, have been described in the AASLD guideline (Figure S1) [1, 11]. Additionally, the AASLD and Baveno VII guidelines specify the use of balloon tamponade (BT), self‐expandable metal stents (SEMS), and TIPS when hemostasis is difficult [1, 11]. However, Japanese guidelines do not provide specific recommendations for managing such cases (Figure S1) [9, 10].

For the primary prevention of esophageal varices, in Japan, treatment is indicated for patients with F2 or larger varices or those with a positive red color sign (RC). If severe liver damage (Child‐Pugh class C [CP‐C], total bilirubin ≥4 mg/dL) or advanced liver cancer complications (portal vein tumor thrombosis Vp3/Vp4) are absent, EIS is selected; otherwise, EVL is preferred (Figure S2) [9, 10].

In contrast, the AASLD and Baveno VII guidelines indicate treatment for patients with a positive RC or F2 or larger varices (CTP‐C is also included as an indication in the AASLD guidelines). The primary treatment options in these guidelines are EVL and NSBB (Figure S2) [1, 11]. Compared to Europe and the United States, endoscopic treatment plays a more central role in the primary and secondary prevention of esophageal varices in Japan.

### 1.2 Difficult Cases for Endoscopic Treatment

In this study, we reviewed treatment approaches for esophageal varices that are difficult to manage endoscopically, categorizing them into IVR, surgical treatment, and medical treatment (Table 1).

A search was conducted on PubMed for “esophageal varices” and “esophageal varices bleeding” and on Igaku Chuo Zasshi for “esophageal varices” and “bleeding esophageal varices” from 1980 to 2024. Tables 4 (Case Reports) and 5 (Original Articles) show 19 reported cases (peer‐reviewed).

**TABLE 4:** Literature review of difficult cases for endoscopic treatment of esophageal varices (case reports).

No.	Year	Authors	Age/Sex	Etiology	Pipeline type (±)	Emergency/Elective	Treatment details	Treatment
1	1991	Shimakawa [31]	51/M	Alcohol	+	Elective	Esophageal transection (PSE performed before surgery)	EIS was performed five times without success. PSE was performed before esophageal transection.
2	1997	Umezawa [32]	42/M	HCV	+	Elective	TIPS→EIS	After nine attempts of EIS were unsuccessful, TIPS was performed. One year later, rebleeding occurred, and the shunt was re‐established, followed by seven attempts of EIS, which were successful.
3	1997	Umezawa [32]	63/F	HCV	+	Elective	TIPS→EIS	Pipeline varices and perforators made EIS difficult. TIPS was performed first, followed by five sessions of EIS.
4	2006	Imai [29]	64/F	HCV	+	Elective	ETA→ EIS	Three EO method sessions were unsuccessful. Successful hemostasis was achieved with N‐butyl‐2‐cyanoacrylate injection + EO method.
5	2008	Caronna [33]	58/‐	Non‐cirrhotic	–	Emergency	Esophageal transection →Percutaneous portal vein stent placement	Endoscopic hemostasis was difficult; esophageal transection was performed but failed. Hemostasis was achieved by percutaneous portal vein stenting.
6	2012	Phillip [34]	‐/‐	Primary myelofibrosis	–	Emergency	TIPS	Endoscopic hemostasis was difficult, but hemostasis was achieved using TIPS.
7	2020	Chikamori [35]	74/F	NASH	–	Elective	Stepwise PSE→EISL	Two unsuccessful EIS attempts; stepwise PSE was performed (second PSE after 2 months), followed by EISL 4 months later.
8	2022	Chikamori [36]	49/M	Alcohol	–	Emergency	Hybrid surgery of EISL and PSE	After initial PSE and rebleeding, EVL was performed. Emergency hybrid surgery under general anesthesia involved EISL followed by PSE.
9	2022	Chikamori [37]	64/M	Wilson's disease	–	Emergency	PTO	Stepwise PSE was performed; EVL was attempted but bleeding occurred after EVL. PTO was performed for hemostasis.
10	2022	Yokoyama [38]	29/M	Extrahepatic portal vein obstruction	+	Emergency	TIO→EIS	EIS failed and puncture site bleeding was uncontrollable with balloon tamponade. EVL was insufficient, so emergency TIO embolization was performed, followed by EIS due to residual blood flow.
11	2022	Fujii [39]	34/M	AIH+HCV	–	Elective	TIO	CT showed dilated paraesophageal varices; EIS was deemed difficult, and TIO was performed.
12	2023	Hosokawa [40]	48/M	Alcohol	–	Emergency	PTO	EVL was difficult (aspiration issue); hemostasis was achieved with PTO.
13	2023	Chikamori [41]	53/M	Alcohol+HCV	+	Elective	PSE→EISML	After initial EVL for rupture, pipeline esophageal varices and splenomegaly prompted stepwise PSE. After PSE (time of second PSE unclear), EISML was performed. Due to high HVPG, a third PSE was added.

Abbreviations: AIH, autoimmune hepatitis; EIS, endoscopic injection sclerotherapy; EISL, endoscopic injection sclerotherapy with ligation; EISML, endoscopic injection sclerotherapy with multiple ligations; EO, ethanolamine oleate; ETA, endoscopic therapy with tissue adhesives; EVL, endoscopic variceal ligation; HCV, hepatitis C virus; HVPG, hepatic venous pressure gradient; NASH, non‐alcoholic steatohepatitis; PSE, partial splenic embolization; PTO, percutaneous transhepatic obliteration; TIO, transileocolic vein obliteration; TIPS, transjugular intrahepatic portosystemic shunt.

**TABLE 5:** Literature review of difficult cases for endoscopic treatment of esophageal varices (Original Articles).

No.	Year	Authors	Emergency/Elective	Treatment	Treatment details
1	1989	Jenkins [42]	Emergency	Esophageal transection	Esophageal transection was performed in 15 patients where hemostasis was difficult with conservative (including endoscopic) treatment. Eleven of the 15 patients died in hospital (details unknown).
2	1995	Bizollon [43]	Emergency	TIPS	TIPS was performed in 11 patients with difficult endoscopic hemostasis, achieving successful hemostasis.
3	2014	Cho [44]	Emergency	PTO	Due to an insufficient environment for endoscopic treatment, PTO was performed in seven patients. Hemostasis was achieved, but two patients died from disseminated intravascular coagulation and massive gastrointestinal bleeding two days after the procedure.
4	2017	Buechter [45]	Emergency	PSE	PSE was performed in three patients with difficult endoscopic treatment, achieving hemostasis.
5	2023	Pavel [46]	Emergency	PSE	PSE was performed in seven patients with difficult endoscopic treatment, and hemostasis was achieved.
6	2024	Iwasaki [47]	Elective	Gastropancreatic fold division/splenectomy	Surgical intervention was performed in five patients who did not improve after one or more endoscopic or interventional radiology treatments. Four patients underwent gastropancreatic fold division, and one underwent splenectomy.

Abbreviations: DIC, disseminated intravascular coagulation; PSE, partial splenic embolization; PTO, percutaneous transhepatic obliteration; TIPS, transjugular intrahepatic portosystemic shunt.

Of the 19 cases, 16 involved IVR (four TIPS, seven partial splenic embolization (PSE), two percutaneous transhepatic obliteration [PTO], two TIO, and one percutaneous portal vein stent placement), four involved surgical treatment (three esophageal transections and one splenectomy and gastropancreatic fold division), and one involved medical treatment (ETA with NBCA).

Until the 2000s, esophageal transections and TIPS were common, but their frequency has decreased since the 2010s. In contrast, there have been numerous reports of IVR procedures such as PSE, PTO, and TIO, or a combination of IVR and endoscopic treatments, with many reports from Japan. Surgical treatments have also included minimally invasive approaches, such as GPFD.

### 1.3 Interventional Radiology

IVR for portal hypertension can be classified into two approaches. The first is portal vein decompression, which includes TIPS and PSE. The second is collateral circulation embolization, such as PTO or transileocolic vein obliteration (TIO) (Table 1) [39].

### 1.4 Transjugular Intrahepatic Portosystemic Shunt

**FIGURE 2:**
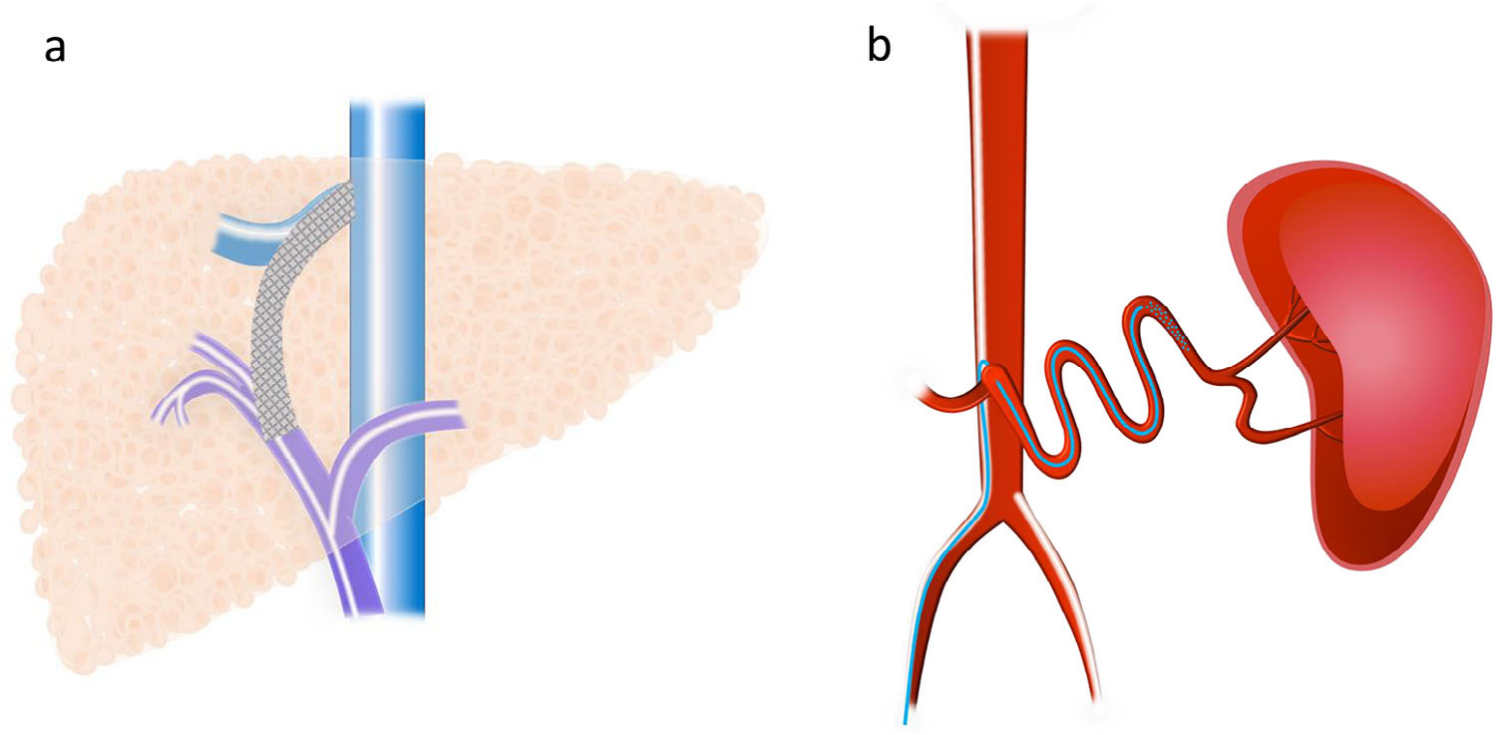
Portal decompression techniques: (a) Transjugular intrahepatic portosystemic shunt (TIPS). A catheter is inserted into the hepatic vein via a transjugular approach, followed by a puncture from the hepatic vein into the portal vein. An expandable polytetrafluoroethylene‐covered stent graft is then inserted to create a connection between the portal and hepatic veins. TIPS reduces portal hypertension and prevents esophageal variceal bleeding. (b) Partial splenic embolization (PSE). PSE is performed via the femoral artery approach using a microcatheter to access the peripheral branches of the splenic blood vessels. Embolization is then performed using metal coils or a gelatin sponge. PSE reduces portal vein pressure and splenic vein blood flow.

TIPS is a procedure used to manage complications of portal hypertension by creating a portosystemic shunt that percutaneously connects an intraparenchymal branch of the portal vein to the hepatic vein (Figure 2a) [48]. TIPS is typically performed via a transjugular approach under general anesthesia or deep sedation. A catheter is inserted into the hepatic vein, followed by a puncture from the hepatic vein into the portal vein. An expandable polytetrafluoroethylene‐covered stent graft is then inserted to create a connection between the portal and hepatic veins [49].

The AASLD and Baveno VII guidelines outline the following roles for TIPS: (1) early TIPS (within 72 hours after endoscopy) to prevent rebleeding of esophageal varices after medical treatment, and (2) salvage therapy for esophageal variceal bleeding that cannot be controlled by medical treatment [49, 50, 51, 52, 53]. Additionally, TIPS is not recommended for the primary prevention of esophageal variceal bleeding [49].

In patients with acute esophageal variceal bleeding, early TIPS has been reported to reduce the risk of rebleeding and death compared to standard treatment (a combination of endoscopic and drug therapy) or non‐early TIPS [49, 54]. The AASLD guidelines recommend early TIPS as secondary prevention for patients with CTP class B (scores 8–9) with active bleeding or CTP class C (score 10–13) (Supplementary Figure S1) [11]. However, some studies suggest that early TIPS does not provide a significant survival benefit compared to standard treatment [55]. Therefore, selecting high‐risk patients who are likely to benefit from early TIPS is crucial [56, 57, 58].

For refractory variceal bleeding, the AASLD and Baveno VII guidelines recommend salvaging TIPS after bridging therapy with BT or SEMS (Figure S1) [1, 50]. Although bleeding control is achieved in 80%–100% of cases, the 6‐week mortality rate remains high, with common causes of death including liver failure, infection, and renal failure [59, 60, 61, 62].

Although TIPS is recommended by the European Society of Gastrointestinal Endoscopy (ESGE), AASLD, Baveno VII, and other guidelines, it is not included in the Japanese guidelines. In Japan, TIPS is a self‐paid procedure and is not commonly used for treating refractory esophageal varices.

### 1.5 Partial Splenic Embolization

Portal hypertension alters blood flow to the spleen, causing splenomegaly [63], which further increases portal vein blood flow, exacerbating portal hypertension and contributing to treatment resistance [64].

PSE is an endovascular treatment used to alleviate portal hypertension in patients with acute or recurrent variceal bleeding. PSE is performed via the femoral artery approach, using a microcatheter to access the peripheral branches of the splenic blood vessels. Embolization is then carried out using metal coils or a gelatin sponge (Figure 2b) [45, 65]. PSE not only increases platelet counts but also reduces portal vein pressure, splenic vein blood flow, and the spleen‐to‐liver volume ratio [35, 66–68]. Complications of PSE include splenic abscess, pneumonia, hematoma, pancreatic infarction, and sepsis [69].

ESGE and Baveno VII do not include PSE in the treatment algorithm for esophageal varices [50, 70]. Conversely, the Japanese guidelines recommend PSE in cases where severe portal hypertension is suspected, such as cases resistant to endoscopic treatment or those with splenomegaly [9, 10]. When medical therapy and endoscopic treatment have been unsuccessful and TIPS is either not feasible or contraindicated, PSE has been selected as a rescue treatment for gastroesophageal variceal bleeding, with successful outcomes reported [45, 46]. Furthermore, a hybrid treatment combining endoscopic therapy and PSE for esophagogastric varices has also been reported and may play an important role in the future management of esophageal variceal bleeding [36, 71].

### 1.6 Percutaneous Transhepatic Obliteration

**FIGURE 3:**
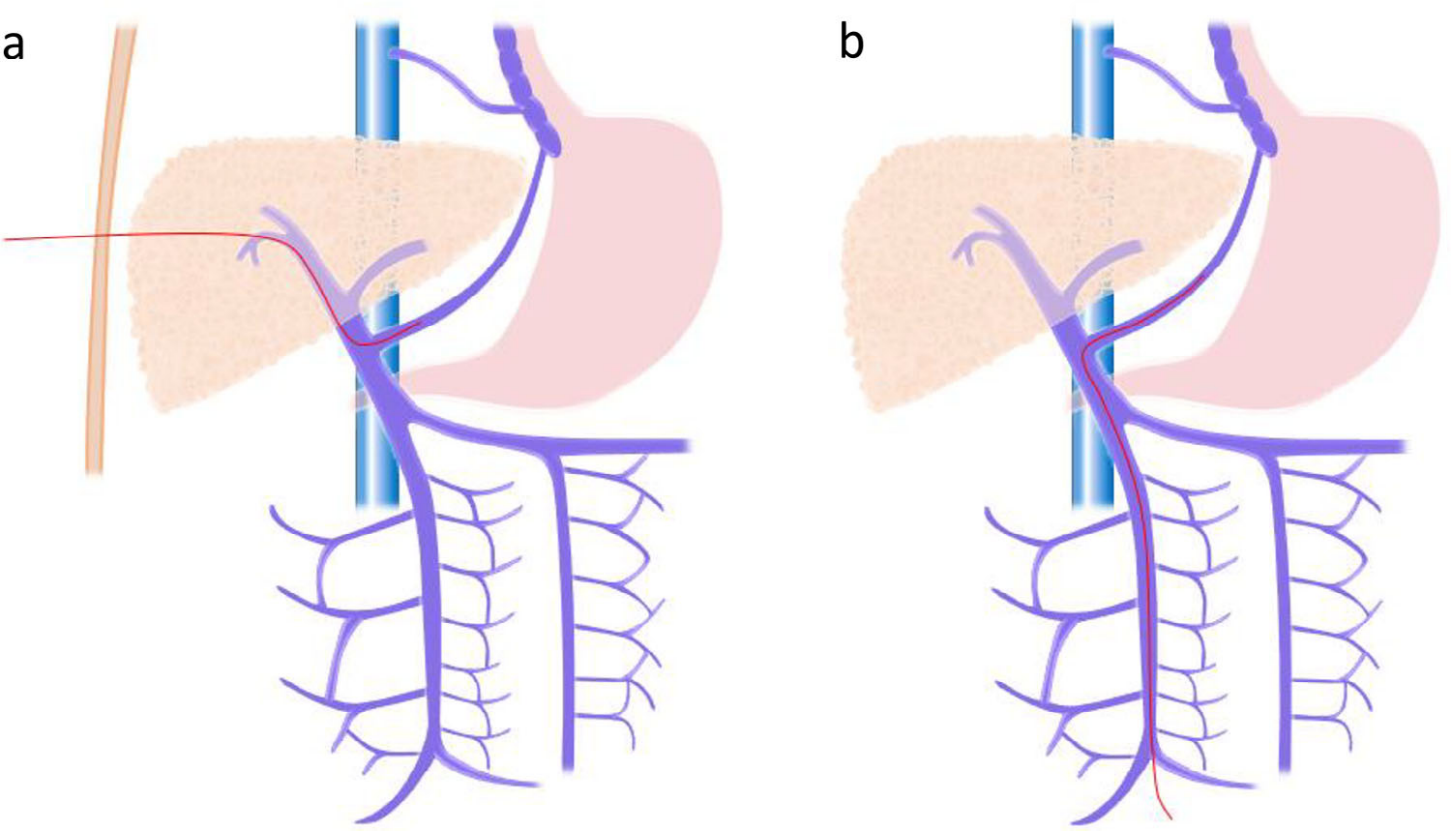
Collateral embolization techniques: (a) Percutaneous transhepatic obliteration (PTO). PTO is a procedure in which a catheter is inserted percutaneously and transhepatically into the portal vein to embolize collateral circulation branching from the portal vein. (b) Transileocolic vein obliteration (TIO). TIO is a procedure in which the ileocolic vein is exposed through laparotomy and a catheter is inserted antegrade through the ileal vein to embolize the varices.

PTO is a procedure in which a catheter is inserted percutaneously and transhepatically into the portal vein to embolize collateral circulation branching from the portal vein (Figure 3a) [40, 72]. Previously, PTO had the advantage of being less invasive and causing fewer complications than surgery. However, with the advent of EVL, which is even less invasive and associated with fewer complications [40], EVL has become the first‐line treatment for ruptured esophageal varices [40]. PTO is contraindicated in cases of severe hepatic atrophy, ascites, and tumorous lesions in the liver [39].

In a prospective randomized controlled trial, EIS was superior to PTO for the long‐term control of variceal bleeding [73]. Currently, PTO is considered an acute treatment for stopping bleeding. When endoscopic treatment or TIPS is not feasible, PTO may be a treatment option.

### 1.7 Transileocolic Vein Obliteration

TIO is a procedure in which the ileocolic vein is exposed through laparotomy and a catheter is inserted antegrade through the ileal vein to embolize the varices (Figure 3b). The indications for TIO in esophageal varices include “cases that are difficult to treat with endoscopic therapy” and “cases that cannot be treated with PTO.” [39] As TIO alone is associated with a high risk of rebleeding, combining TIO with endoscopic therapy can increase the success rate of hemostasis [74].

### 1.8 Surgical Therapy

Surgical treatments are classified into “direct surgery,” “selective shunting,” and “systemic shunting” (Table 1). After Whipple established the portal vein obstruction theory in 1945, portacaval shunt (PCS) became the mainstream approach [14]. However, due to the frequent occurrence of Eck fistula syndrome (postoperative hepatic encephalopathy and liver failure) after surgery, PCS was no longer performed in Japan in the 1960s [14].

In the 1960s, direct surgery and selective shunting were developed. In 1964, Hassab et al. performed the Hassab operation, which involved esophageal transection, splenectomy, and occlusion of blood flow to the upper esophagus and stomach [75, 76]. In 1967, Sugiura et al. reported a transthoracic and transabdominal esophageal transection (esophageal transection + splenectomy + occlusion of blood flow to the upper esophagus and stomach) [77], and in the same year, Warren reported the distal splenorenal shunt [78].

For example, regarding the indications for the Hassab operation, Yoshioka et al. defined them as “cases difficult to treat with endoscopy,” “cases up to Child‐Pugh A/B,” “cases with severe hypersplenism,” and “cases aged 65 years or younger in principle” [79]. Surgical treatments have stricter age restrictions than interventional vein treatments and are more invasive, leading to a decrease in the number of surgical treatments performed each year.

The Baveno VII guidelines state that, in cases where hemostasis is difficult due to esophageal varices and when nonsurgical treatments fail or IVR cannot be performed, surgical treatment is listed as an option. However, the guidelines lack a detailed description of which surgical procedure is best [1, 50]. The AASLD guidelines do not mention surgical treatment [11]. The Japanese guidelines list splenectomy as a surgical treatment [9, 10]. As a result, the indications for treatment and the choice of surgical procedure are currently left to the discretion of each medical institution. Recently, less invasive treatments, such as laparoscopic Hassab surgery and gastropancreatic fold division (GPFD) [47], have been reported, with further developments expected in the future.

### 1.9 Internal Medicine Treatment

#### 1.9.1 Self‐expandable Metal Stents

The use of SEMS or BT is recommended as a temporary measure for treating esophageal variceal bleeding when endoscopic hemostasis is unsuccessful [11, 47, 80]. SEMS has a high success rate in immediate bleeding control, with rates reaching up to 90.6% [81].

A meta‐analysis regarding the use of SEMS for refractory variceal bleeding reported that mortality and rebleeding rates were higher than those for TIPS [82]. The ESGE guidelines consider SEMS a bridge therapy to TIPS [83]. Randomized controlled trials have shown that SEMS is more effective than BT in controlling esophageal variceal bleeding [84], and the ESGE clearly states that SEMS is preferred over BT as a bridge therapy in cases of persistent esophageal variceal bleeding [70].

SEMS has a lower incidence of complications compared to the SB tube [81]. After stent placement, oral intake becomes possible, allowing time for liver function improvement and bridging the gap to TIPS or orthotopic liver transplantation [85]. Rodge et al. reported the duration of SEMS placement to be a maximum of 14 days [86], while Songtanin et al. reported a range of 2–17.5 days [81]. SEMS is used as a bridge therapy until the next treatment. Adverse events after stent placement include rebleeding (16.6%), stent ulceration (6.8%), and stent migration (18.2%) [81].

#### 1.9.2 Balloon Tamponade

Similar to SEMS, BT is used as a temporary measure to treat esophageal variceal bleeding when endoscopic hemostasis fails [80]. BT aims to achieve temporary hemostasis by directly compressing the bleeding varices using the SB tube or Minnesota tube [87]. The difference between the two tubes is that the SB tube has suction ports for gastric contents only, while the Minnesota tube has suction ports for both the stomach and esophagus [87].

The SB tube was first introduced by Sengstaken and Blakemore in 1950 [88]. The control rate of esophageal variceal bleeding with the SB tube has been reported to be 91.5% [86]. The duration of BT placement is limited to 24 hours, and like SEMS, its main role is to provide temporary hemostasis until the next treatment [47, 80]. Tube placement prevents patients from swallowing saliva, increasing the risk of dysphagia and aspiration pneumonia [89]. If the tube is left in place for more than 24 hours, serious complications such as esophageal perforation and ischemic necrosis of the mucosa may occur in 6%–20% of cases, and these complications are thought to occur more frequently in inexperienced hands [86, 90].

#### 1.9.3 Non‐selective β‐Blocker

NSBBs are a cornerstone of treatment for portal hypertension as they reduce the risk of variceal bleeding and rebleeding while providing a palliative effect on portal vein pressure. The European Association for the Study of the Liver, AASLD, and Baveno VII guidelines recommend the use of NSBBs for the primary and secondary prevention of variceal bleeding in cirrhotic patients with gastroesophageal varices [91].

Types of NSBBs used to treat portal hypertension include propranolol, nadolol, and carvedilol [1]. Conventional NSBBs such as propranolol and nadolol block β1 and β2 adrenergic receptors. Carvedilol, in addition to blocking β1 and β2 adrenergic receptors, also has α1 adrenergic receptor blockade, which further reduces intrahepatic vascular resistance [92, 93]. Carvedilol is also effective in reducing the HVPG and preventing decompensation. It is better tolerated than conventional NSBBs (propranolol, nadolol) and improves survival rates [1, 50]. The AASLD has described the mechanism and dosage of each drug (Table S1) [11].

Carvedilol is recommended for the treatment of portal hypertension, starting at a low dose (6.25 mg/day) and gradually increasing to a maximum of 12.5 mg/day, depending on the patient's tolerance [94]. Its side effects are related to its β1/β2 blocking activity and include contraindications in patients with asthma, COPD, severe bradycardia, or atrioventricular block [94].

#### 1.9.4 Vasoactive Agents

Vasoactive drugs used for acute variceal hemorrhage (AVH) are classified into (1) somatostatin, (2) octreotide (a somatostatin analog), and (3) terlipressin (a vasopressin analog). These vasoactive agents work by vasoconstricting the splanchnic circulation, thereby reducing portal vein pressure [95, 96].

Previously, vasopressin was also used, but due to its high risk of cardiovascular adverse events, it is no longer recommended for AVH patients [95]. Vasoactive drugs should be initiated before diagnostic and therapeutic endoscopy for variceal bleeding and continued for 2–5 days after endoscopic hemostasis to prevent early rebleeding [50, 95, 97, 98]. The American Gastroenterological Association (AGA) and ESGE recommend the use of terlipressin, octreotide, and somatostatin for AVH [70, 95]. The Baveno VII guideline also recommends these agents [50]. However, vasoactive drugs are not routinely indicated in Japan, and supporting evidence is lacking. The AGA has described standard regimens for each drug (Table S2) [95].

#### 1.9.5 Others

We think that patients with esophageal varices and low platelet counts are difficult to treat.

The Japanese liver cirrhosis guidelines, AASLD, and Baveno VII do not state specific platelet count treatment targets for esophageal varices treatment. However, the Society for Interventional Radiology (USA) recommends a platelet count over 30 × 10^9^/L as a safe threshold for performing high‐risk bleeding procedures [99]. Furthermore, the American College of Gastroenterology recommends a platelet count over 50 × 10^9^/L as a safe threshold for performing high‐risk bleeding procedures [100].

## 2 Discussion

Our review highlights regional differences in treatment approaches and suggests that multidisciplinary collaboration can help overcome these challenges.

Regarding IVR, the treatment policies in Japan and Western countries differ as outlined below. First, Western guidelines include recommendations for TIPS. In contrast, Japanese guidelines do not mention TIPS as it is considered a private medical treatment in Japan, and the number of facilities capable of performing TIPS is very limited. Second, Japanese guidelines specify PSE as a treatment option, whereas European and American guidelines do not. Third, in Japan, there have been numerous reports of IVR treatments involving not only PSE but also PTO and TIO. Therefore, the choice of IVR treatment varies by country and region.

Surgical treatment is not specified in the Japanese or AASLD guidelines, and the Baveno VII guidelines mention it only as a “measure when hemostasis is difficult with nonsurgical treatment.” In current esophageal variceal treatment, surgical intervention is generally considered on a case‐by‐case basis by each medical institution for varices that are difficult to control with endoscopic treatment or IVR, while also considering the invasiveness of the procedure.

Regarding internal medicine treatment, the Japanese guidelines mention only EVL and BT using an SB tube for ruptured esophageal varices. In contrast, the AASLD and Baveno VII guidelines list multiple treatment options, such as EVL, SEMS, BT, and ETA. In a setting where IVR or surgical intervention is not feasible, medical treatment becomes essential, highlighting the need to explore and validate various therapeutic approaches.

In the summary of difficult endoscopic treatment cases, earlier reports frequently described the use of esophageal transection and TIPS for emergency hemostasis. However, more recent reports highlight the increasing use of PSE and PTO.

For elective cases, pipeline esophageal varices and well‐developed paraesophageal veins were commonly observed. In these cases, a combination of IVR and endoscopic treatment, such as PSE and TIO, has been frequently reported.

A comparison of the guidelines from Japan, Europe, and the United States revealed differences in the recommended treatments for esophageal varices. By referring to international guidelines and reports on difficult‐to‐treat cases, multidisciplinary cooperation can be enhanced to improve outcomes in challenging cases.

## Conflicts of Interest

The authors declare no conflicts of interest.

## Ethics Statement

**Registry and Registration Number**: This retrospective study was approved by the Ethics Review Committee of Yamagata University School of Medicine (Approval number: 2019–316).

## Consent

All patients provided informed consent for the use of their clinical information for research.

## Supporting information




**Supporting Fig. 1**: Guidelines for the management of acute variceal bleeding and secondary prophylaxis in Japan, AASLD, and Baveno IV. (supporting information legend). EVL, endoscopic variceal ligation; SB tube, Sengstaken–Blakemore tube; CTP‐C, child‐turcotte‐pugh grade C; T‐Bil, total bilirubin; Vp, vascular permeation; EIS, endoscopic injection sclerotherapy; AS, aethoxysklerol; APC, argon plasma coagulation; Hb, hemoglobin; CTRX, ceftriaxone; NSBB, non‐selective β‐blockers; TIPS, transjugular intrahepatic portosystemic shunt; BSC, best supportive care; ETA, endoscopic therapy with tissue adhesives; HP, hemostatic powders; IVR interventional radiology.

**Supporting Fig. 2**: Guidelines for primary prophylaxis of esophageal varices in Japan, AASLD, and Baveno IV. (supporting information legend). RC, red color sign; F, form: F1, small/straight; F2, enlarged/tortuous; F3, large/coil‐shaped; CTP‐C, child‐turcotte‐pugh grade C; T‐Bil, total bilirubin; Vp, vascular permeation; EIS, endoscopic injection sclerotherapy; EVL, endoscopic variceal ligation; AS, aethoxysklerol; APC, argon plasma coagulation; NSBB, non‐selective β‐blockers.

**Supporting Table 1**: Types of NSBBs used for treating portal hypertension.
**Supporting Table 2**: Types of Vasoactive agents used to reduce portal vein pressure.
