# Supplementary figures and images for: Management Strategies for Refractory Esophageal Varices
Source: DEN Open. 2025 Jun 19;6(1):e70155. doi: 10.1002/deo2.70155 (PMC12177223; doi:10.1002/deo2.70155)

Supplementary Figure 1

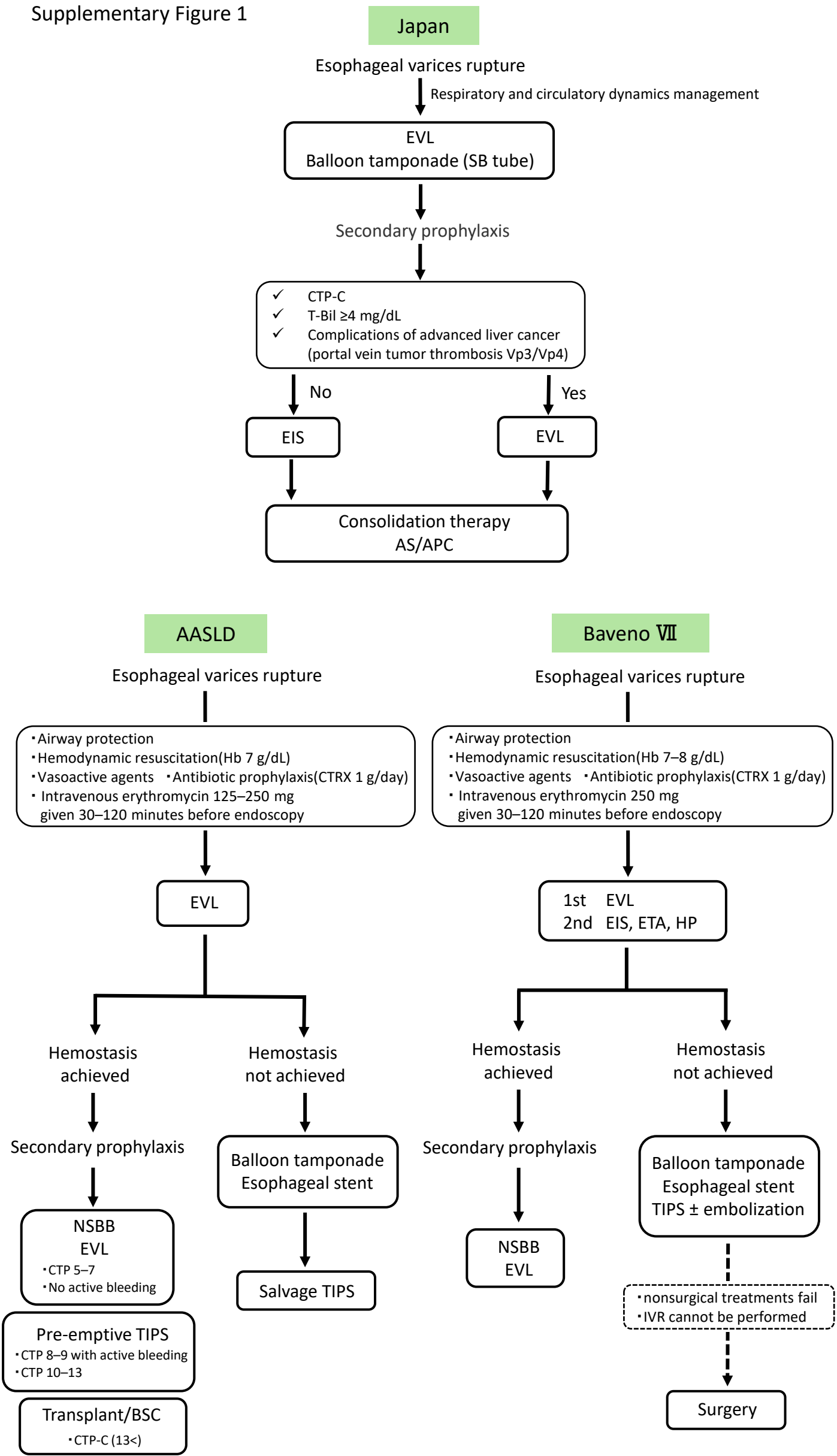

Supplement: Supplementary file 1 — Supporting Fig. 1: Guidelines for the management of acute variceal bleeding and secondary prophylaxis in Japan, AASLD, and Baveno IV. (supporting information legend). EVL, endoscopic variceal ligation; SB tube, Sengstaken–Blakemore tube; CTP‐C, child‐turcotte‐pugh grade C; T‐Bil, total bilirubin; Vp, vascular permeation; EIS, endoscopic injection sclerotherapy; AS, aethoxysklerol; APC, argon plasma coagulation; Hb, hemoglobin; CTRX, ceftriaxone; NSBB, non‐selective β‐blockers; TIPS, transjugular intrahepatic portosystemic shunt; BSC, best supportive care; ETA, endoscopic therapy with tissue adhesives; HP, hemostatic powders; IVR interventional radiology. [file DEO2-6-e70155-s002.pdf]

Supplementary Figure 2

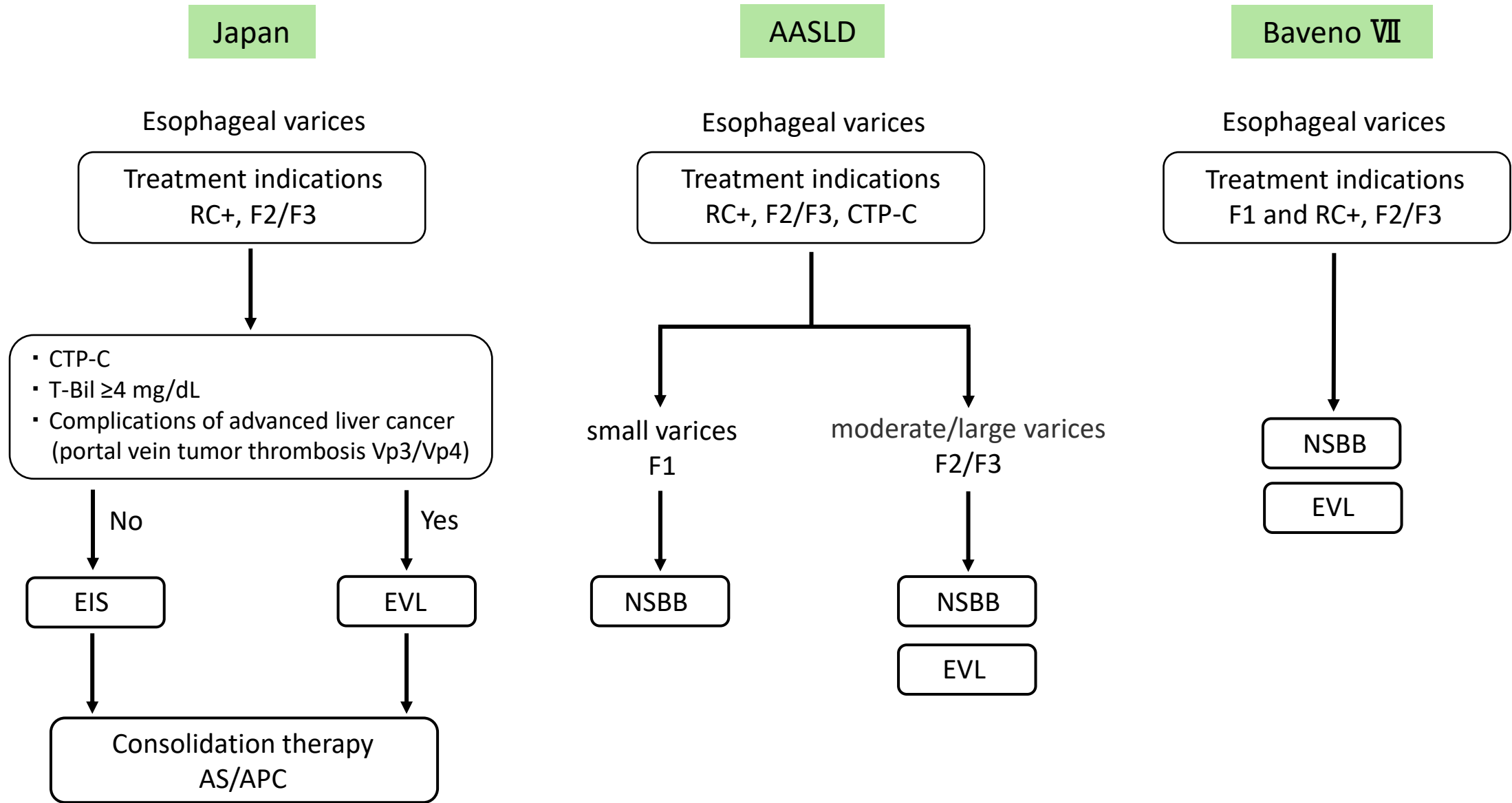

Supplement: Supplementary file 2 — Supporting Fig. 2: Guidelines for primary prophylaxis of esophageal varices in Japan, AASLD, and Baveno IV. (supporting information legend). RC, red color sign; F, form: F1, small/straight; F2, enlarged/tortuous; F3, large/coil‐shaped; CTP‐C, child‐turcotte‐pugh grade C; T‐Bil, total bilirubin; Vp, vascular permeation; EIS, endoscopic injection sclerotherapy; EVL, endoscopic variceal ligation; AS, aethoxysklerol; APC, argon plasma coagulation; NSBB, non‐selective β‐blockers. [file DEO2-6-e70155-s003.pdf]
