# Supplementary material for: Management Strategies for Refractory Esophageal Varices
Source: DEN Open. 2025 Jun 19;6(1):e70155. doi: 10.1002/deo2.70155 (PMC12177223; doi:10.1002/deo2.70155)
Supplement: Supplementary file 3 — Supporting Table 1: Types of NSBBs used for treating portal hypertension. Supporting Table 2: Types of Vasoactive agents used to reduce portal vein pressure. [file DEO2-6-e70155-s001.docx]

**Supplementary Table 1.** Types of NSBBs used for treating portal hypertension

| Drug | Mechanism of action | Starting dose | Maximal dose |
| --- | --- | --- | --- |
| Propranolol | Decreases cardiac output through beta-1 blockade (reducing heart rate and contractility) and beta-2 bl blockade (causing splanchnic vasoconstriction). | 20–40 mg twice daily | Without ascites: 320 mg/day;  With ascites: 160 mg/day |
| Nadolol | Causes splanchnic vasoconstriction through beta-2 blockade, allowing unopposed alpha-adrenergic vasoconstriction. | 20–40 mg at bedtime | Without ascites: 160 mg/day;  With ascites: 80 mg/day |
| Carvedilol | In addition to above actions, it decreases intrahepatic vascular resistance through anti-alpha-adrenergic activity. | 6.25 mg once daily | 12.5 mg/day (higher doses may be used for nonhepatic indications) |

**Supplementary Table 2.** Types of Vasoactive agents used to reduce portal vein pressure

| Drug | Mechanism of action |
| --- | --- |
| Octreotide (somatostatin analogue) | 50 μg IV bolus, followed by continuous IV infusion at 50 μg/h; additional IV boluses can be given for ongoing bleeding |
| Somatostatin | 250 μg IV bolus, followed by continuous IV infusion at 250–500 μg/h; additional IV boluses can be given for ongoing bleeding |
| Terlipressin (vasopressin analogue) | Initial 48 h: 2 mg IV every 4 h until bleeding is controlled.  Maintenance: 1 mg IV every 4 h |
